# Poly[[hemi-μ_4_-oxalato-hemi-μ_2_-oxalato-bis­(μ_3_-pyrazine-2-carboxyl­ato)erbium(III)silver(I)] monohydrate]

**DOI:** 10.1107/S1600536809029742

**Published:** 2009-07-31

**Authors:** Ling-Zhi Zhao, Rui-Xia He, Qiu-Gui Zhong, Rong-Hua Zeng, Dong-Sheng Lu

**Affiliations:** a School of Chemistry and Environment, South China Normal University, Guangzhou 510006, People’s Republic of China; b Key Laboratory of the Technology of Electrochemical Energy Storage and Power Generation in Guangdong Universities, South China Normal University, Guangzhou 510006, People’s Republic of China

## Abstract

The asymmetric unit of the title complex, {[AgEr(C_5_H_3_N_2_O_2_)_2_(C_2_O_4_)]·H_2_O}_*n*_, contains one Er^III^ atom, one Ag^I^ atom, two pyrazine-2-carboxyl­ate (pyc) ligands, two half oxalate ligands (each lying on an inversion center) and one uncoordinated water mol­ecule. The Er^III^ atom is coordinated by two O atoms and two N atoms from two pyc ligands, one O atom from a third pyc ligand and four O atoms from two oxalate ligands in a distorted monocapped square-anti­prismatic geometry. The Ag^I^ atom is coordinated by two N atoms from two pyc ligands, one O atom from a third pyc ligand and one O atom from one oxalate ligand. The crystal structure exhibits a three-dimensional heterometallic polymeric network. O—H⋯O hydrogen bonding between the uncoordinated water mol­ecule and carboxyl­ate O atoms is observed.

## Related literature

For general background to lanthanide–transition heterometallic complexes, see: Deng *et al.* (2008 ▶); Wang *et al.* (2006 ▶); Zhou *et al.* (2006 ▶).
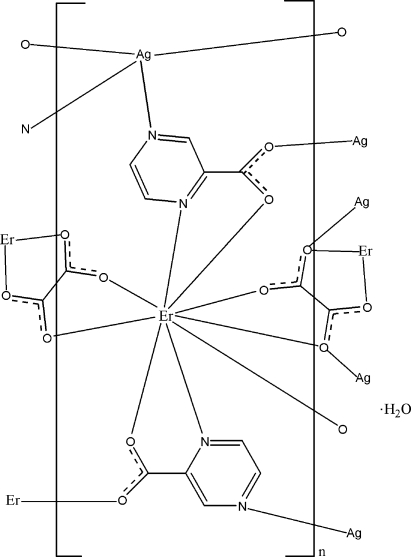


## Experimental

### 

#### Crystal data

[AgEr(C_5_H_3_N_2_O_2_)_2_(C_2_O_4_)]·H_2_O
*M* *_r_* = 627.35
Monoclinic, 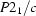

*a* = 10.0482 (6) Å
*b* = 18.3968 (11) Å
*c* = 8.0371 (5) Å
β = 95.397 (1)°
*V* = 1479.11 (16) Å^3^
*Z* = 4
Mo *K*α radiation
μ = 7.02 mm^−1^
*T* = 296 K
0.22 × 0.20 × 0.19 mm

#### Data collection

Bruker APEXII CCD diffractometer
Absorption correction: multi-scan (*SADABS*; Sheldrick, 1996 ▶) *T*_min_ = 0.307, *T*_max_ = 0.349 (expected range = 0.232–0.263)
7533 measured reflections
2649 independent reflections
2450 reflections with *I* > 2σ(*I*)
*R*_int_ = 0.019

#### Refinement

*R*[*F* ^2^ > 2σ(*F* ^2^)] = 0.021
*wR*(*F* ^2^) = 0.050
*S* = 1.04
2649 reflections
244 parameters
12 restraints
H-atom parameters constrained
Δρ_max_ = 1.43 e Å^−3^
Δρ_min_ = −1.14 e Å^−3^

### 

Data collection: *APEX2* (Bruker, 2007 ▶); cell refinement: *SAINT* (Bruker, 2007 ▶); data reduction: *SAINT*; program(s) used to solve structure: *SHELXS97* (Sheldrick, 2008 ▶); program(s) used to refine structure: *SHELXL97* (Sheldrick, 2008 ▶); molecular graphics: *SHELXTL* (Sheldrick, 2008 ▶) and *Mercury* (Macrae *et al.*, 2006 ▶); software used to prepare material for publication: *SHELXTL*.

## Supplementary Material

Crystal structure: contains datablocks I, global. DOI: 10.1107/S1600536809029742/hy2207sup1.cif

Structure factors: contains datablocks I. DOI: 10.1107/S1600536809029742/hy2207Isup2.hkl

Additional supplementary materials:  crystallographic information; 3D view; checkCIF report

## Figures and Tables

**Table 1:** Selected bond lengths (Å)

Er1—O4	2.333 (3)
Er1—O7	2.367 (3)
Er1—O1	2.385 (3)
Er1—O6^i^	2.387 (3)
Er1—O8^ii^	2.388 (3)
Er1—O2^iii^	2.403 (3)
Er1—O5	2.451 (3)
Er1—N1	2.611 (4)
Er1—N3	2.636 (4)
Ag1—N4^iv^	2.299 (4)
Ag1—O3^v^	2.312 (3)
Ag1—N2	2.368 (4)
Ag1—O5^vi^	2.648 (4)

Symmetry codes: (i) 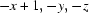; (ii) 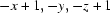; (iii) 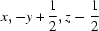; (iv) 
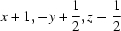
; (v) 
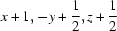
; (vi) 
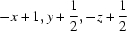
.

**Table 2:** Hydrogen-bond geometry (Å, °)

*D*—H⋯*A*	*D*—H	H⋯*A*	*D*⋯*A*	*D*—H⋯*A*
O1*W*—H1*W*⋯O1	0.86	2.14	2.971 (6)	162

**Fig. 1.**
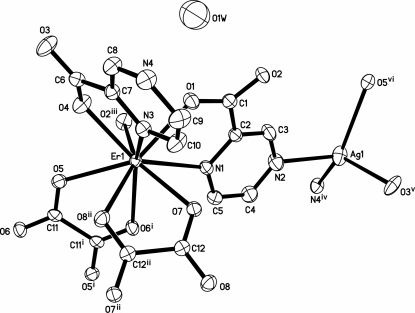
The asymmetric unit of the title compound. H atoms have been omitted for clarity. [Symmetry codes: (i) 1-x, -y, -z; (ii) 1-x, -y, 1-z; (iii) x, 1/2-y, -1/2+z; (iv) 1+x, 1/2-y, -1/2+z; (v) 1+x, 1/2-y, 1/2+z; (vi) 1-x, 1/2+y, 1/2-z.]

**Fig. 2.**
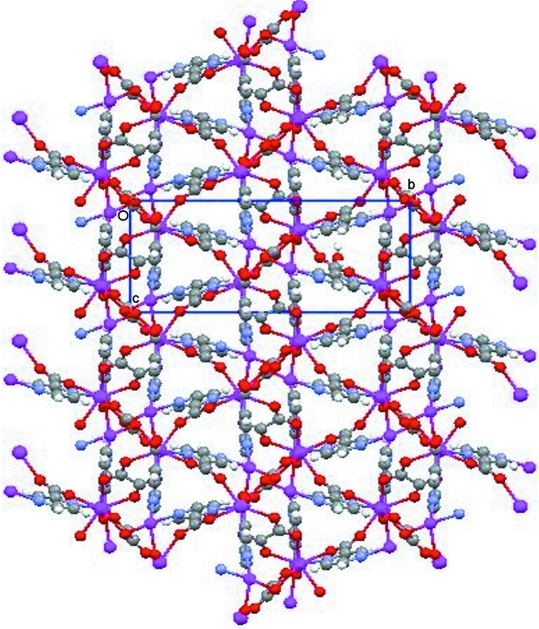
Packing diagram of the title compound.

## supplementary crystallographic information

### Comment

In recent years, many research groups have devoted their work to the design and synthesis of lanthanide–transition heterometallic coordination frameworks with bridging multifunctional organic ligands, not only because of their fascinating topological networks but also due to their potential applications in ion exchange, gas storage, catalysis and luminescence (Wang *et al.*, 2006; Zhou *et al.*, 2006). As a building block, pyrazine-2-carboxylate (pyc) and oxalate are excellent candidates for the construction of heterometallic complexes (Deng *et al.*, 2008). Recently, we obtained the title coordination polymer, which was synthesized under hydrothermal conditions.

The asymmetric unit of the title complex contains one Er^III^ atom, one Ag^I^ atom, two pyc ligands, two half oxalate ligands, each lying on an inversion center, and one uncoordinated water molecule (Fig. 1). The Er^III^ atom is coordinated by two O atoms and two N atoms from two pyc ligands, one O atom from a third pyc ligand and four O atoms from two oxalate ligands. The coordination geometry around the Er^III^ atom can be described as distorted monocapped square-antiprismatic, with Er—O bond distances and O—Er—O bond angles range from 2.333 (3) to 2.451 (3) Å and 66.24 (9) to 147.36 (10)°, respectively (Table 1). The Ag^I^ atom has a distorted tetrahedral coordination geometry, defined by two N atoms from two pyc ligands, one O atom from a third pyc ligand and one O atom from one oxalate ligand. The Ag—N and Ag—O bond distances vary from 2.299 (4) to 2.648 (4) Å. The oxalate ligands bridge the Er atoms to form a zigzag chain. These chains are further interconnected by Ag–pyc subunits into a three-dimensional polymeric network (Fig. 2). O—H···O hydrogen bond involving the carboxylate O atoms of the pyc ligands and uncoordinated water molecules further enhance the stability of the three-dimensional network (Table 2).

### Experimental

A mixture of Er_2_O_3_ (0.183 g, 0.5 mmol), AgNO_3_ (0.170 g, 1 mmol), pyrazine-2-carboxylic acid (0.124 g, 1 mmol), oxalic acid (0.09 g, 1 mmol) and water (10 ml) in the presence of HNO_3_ (0.024 g, 0.385 mmol) was stirred vigorously for 20 min and then sealed in a Teflon-lined stainless steel autoclave (20 ml capacity). The autoclave was heated and maintained at 433 K for 3 d, and then cooled to room temperature at 5 K h^-1^. Colorless block crystals were obtained.

### Refinement

Water H atoms were tentatively located in a difference Fourier map and refined with distance restraints of O—H = 0.86 (1) and H···H = 1.35 Å, and with *U*_iso_(H) = 1.5*U*_eq_(O). H atoms attached to C atoms were positioned geometrically and treated as riding on their parent atoms, with C—H = 0.93 Å and with *U*_iso_(H) = 1.2*U*_eq_(C). The highest residual electron density was found 0.84 Å from Ag1 and the deepest hole 0.73 Å from Ag1.

### Crystal data

**Table:**

[AgEr(C_5_H_3_N_2_O_2_)_2_(C_2_O_4_)]·H_2_O	*F*(000) = 1180
*M**_r_* = 627.35	*D*_x_ = 2.817 Mg m^−^^3^
Monoclinic, *P*2_1_/*c*	Mo *K*α radiation, λ = 0.71073 Å
Hall symbol: -P 2ybc	Cell parameters from 5128 reflections
*a* = 10.0482 (6) Å	θ = 2.2–28.2°
*b* = 18.3968 (11) Å	µ = 7.02 mm^−^^1^
*c* = 8.0371 (5) Å	*T* = 296 K
β = 95.397 (1)°	Block, colorless
*V* = 1479.11 (16) Å^3^	0.22 × 0.20 × 0.19 mm
*Z* = 4	

### Data collection

**Table:**

Bruker APEXII CCD diffractometer	2649 independent reflections
Radiation source: fine-focus sealed tube	2450 reflections with *I* > 2σ(*I*)
graphite	*R*_int_ = 0.019
φ and ω scans	θ_max_ = 25.2°, θ_min_ = 2.0°
Absorption correction: multi-scan (*SADABS*; Sheldrick, 1996)	*h* = −11→12
*T*_min_ = 0.307, *T*_max_ = 0.349	*k* = −22→19
7533 measured reflections	*l* = −9→5

### Refinement

**Table:**

Refinement on *F*^2^	Primary atom site location: structure-invariant direct methods
Least-squares matrix: full	Secondary atom site location: difference Fourier map
*R*[*F*^2^ > 2σ(*F*^2^)] = 0.021	Hydrogen site location: inferred from neighbouring sites
*wR*(*F*^2^) = 0.050	H-atom parameters constrained
*S* = 1.03	*w* = 1/[σ^2^(*F*_o_^2^) + (0.024*P*)^2^ + 4.153*P*] where *P* = (*F*_o_^2^ + 2*F*_c_^2^)/3
2649 reflections	(Δ/σ)_max_ = 0.001
244 parameters	Δρ_max_ = 1.43 e Å^−^^3^
12 restraints	Δρ_min_ = −1.14 e Å^−^^3^

### Fractional atomic coordinates and isotropic or equivalent isotropic displacement parameters (Å^2^)

**Table:**

	*x*	*y*	*z*	*U*_iso_*/*U*_eq_	
Er1	0.364162 (17)	0.105359 (10)	0.24162 (2)	0.01442 (7)	
Ag1	0.85087 (4)	0.42746 (2)	0.39306 (4)	0.03045 (11)	
O1	0.2989 (3)	0.21768 (16)	0.3579 (4)	0.0242 (7)	
O2	0.3493 (3)	0.32200 (16)	0.4921 (4)	0.0235 (7)	
O3	−0.0813 (3)	0.0887 (2)	0.1736 (4)	0.0317 (8)	
O4	0.1381 (3)	0.09487 (18)	0.1482 (4)	0.0250 (7)	
O5	0.3318 (3)	0.01811 (16)	0.0110 (4)	0.0207 (6)	
O7	0.5023 (3)	0.09455 (15)	0.4957 (4)	0.0199 (6)	
N1	0.5506 (3)	0.20455 (19)	0.2760 (4)	0.0190 (8)	
N2	0.7204 (4)	0.3245 (2)	0.3075 (5)	0.0289 (9)	
N3	0.1969 (4)	0.09160 (19)	0.4735 (4)	0.0194 (8)	
N4	−0.0028 (4)	0.0827 (2)	0.6909 (4)	0.0239 (8)	
C1	0.3752 (4)	0.2695 (2)	0.4013 (5)	0.0193 (9)	
C2	0.5118 (4)	0.2678 (2)	0.3374 (5)	0.0195 (9)	
C3	0.5950 (4)	0.3270 (3)	0.3465 (6)	0.0259 (10)	
H3	0.5621	0.3711	0.3818	0.031*	
C4	0.7614 (5)	0.2604 (3)	0.2544 (6)	0.0287 (11)	
H4	0.8491	0.2554	0.2282	0.034*	
C5	0.6765 (4)	0.2009 (3)	0.2372 (6)	0.0255 (10)	
H5	0.7082	0.1573	0.1976	0.031*	
C6	0.0371 (4)	0.0914 (2)	0.2309 (5)	0.0185 (9)	
C7	0.0668 (4)	0.0902 (2)	0.4181 (5)	0.0183 (9)	
C8	−0.0327 (4)	0.0862 (3)	0.5258 (5)	0.0223 (10)	
H8	−0.1219	0.0860	0.4824	0.027*	
C9	0.1265 (4)	0.0855 (3)	0.7468 (5)	0.0267 (10)	
H9	0.1508	0.0845	0.8614	0.032*	
C10	0.2255 (4)	0.0900 (3)	0.6381 (6)	0.0269 (10)	
H10	0.3145	0.0920	0.6818	0.032*	
C11	0.4313 (4)	−0.0108 (2)	−0.0429 (5)	0.0164 (9)	
C12	0.5388 (4)	0.0323 (2)	0.5434 (5)	0.0176 (9)	
O1W	0.0424 (5)	0.2589 (3)	0.4885 (8)	0.0827 (16)	
H1W	0.1096	0.2521	0.4314	0.124*	
H2W	0.0662	0.2368	0.5816	0.124*	
O6	0.4323 (3)	−0.05626 (16)	−0.1587 (4)	0.0212 (7)	
O8	0.6284 (3)	0.01649 (16)	0.6555 (4)	0.0226 (7)	

### Atomic displacement parameters (Å^2^)

**Table:**

	*U*^11^	*U*^22^	*U*^33^	*U*^12^	*U*^13^	*U*^23^
Er1	0.01594 (11)	0.01469 (11)	0.01268 (11)	0.00069 (7)	0.00162 (7)	−0.00034 (7)
Ag1	0.0286 (2)	0.0423 (2)	0.02146 (19)	−0.00868 (16)	0.00783 (14)	−0.00439 (16)
O1	0.0201 (15)	0.0210 (16)	0.0326 (18)	−0.0023 (13)	0.0074 (13)	−0.0060 (14)
O2	0.0281 (17)	0.0210 (16)	0.0212 (16)	0.0042 (13)	0.0014 (13)	−0.0076 (13)
O3	0.0201 (16)	0.059 (2)	0.0152 (16)	0.0027 (15)	−0.0017 (13)	−0.0041 (15)
O4	0.0199 (16)	0.042 (2)	0.0140 (15)	−0.0004 (13)	0.0046 (12)	0.0007 (14)
O5	0.0205 (15)	0.0238 (17)	0.0176 (15)	0.0042 (13)	0.0006 (12)	−0.0029 (13)
O7	0.0255 (16)	0.0166 (15)	0.0169 (15)	0.0009 (12)	−0.0013 (12)	0.0016 (12)
N1	0.0229 (19)	0.0172 (18)	0.0170 (18)	0.0012 (14)	0.0025 (14)	−0.0010 (15)
N2	0.027 (2)	0.028 (2)	0.032 (2)	−0.0040 (17)	0.0074 (17)	−0.0054 (18)
N3	0.0221 (19)	0.0197 (19)	0.0165 (19)	−0.0003 (15)	0.0029 (14)	0.0004 (15)
N4	0.025 (2)	0.032 (2)	0.0160 (19)	0.0015 (16)	0.0052 (15)	0.0012 (16)
C1	0.025 (2)	0.019 (2)	0.013 (2)	0.0028 (18)	0.0001 (17)	0.0025 (18)
C2	0.025 (2)	0.018 (2)	0.015 (2)	0.0029 (17)	0.0000 (17)	0.0000 (17)
C3	0.027 (2)	0.020 (2)	0.031 (3)	−0.0018 (19)	0.0060 (19)	−0.008 (2)
C4	0.020 (2)	0.031 (3)	0.035 (3)	−0.0030 (19)	0.006 (2)	−0.004 (2)
C5	0.022 (2)	0.026 (2)	0.028 (2)	0.0039 (19)	0.0025 (19)	−0.006 (2)
C6	0.021 (2)	0.018 (2)	0.016 (2)	0.0029 (17)	0.0032 (17)	0.0011 (17)
C7	0.020 (2)	0.018 (2)	0.017 (2)	−0.0001 (17)	0.0016 (17)	0.0000 (17)
C8	0.019 (2)	0.032 (3)	0.016 (2)	0.0006 (19)	0.0033 (17)	0.0000 (19)
C9	0.028 (3)	0.039 (3)	0.013 (2)	0.006 (2)	0.0032 (18)	0.004 (2)
C10	0.019 (2)	0.044 (3)	0.018 (2)	0.001 (2)	0.0023 (18)	0.000 (2)
C11	0.023 (2)	0.013 (2)	0.013 (2)	0.0026 (16)	0.0016 (17)	0.0050 (17)
C12	0.019 (2)	0.021 (2)	0.013 (2)	0.0000 (17)	0.0041 (16)	0.0021 (17)
O1W	0.053 (3)	0.067 (3)	0.130 (5)	−0.008 (2)	0.013 (3)	−0.020 (3)
O6	0.0200 (15)	0.0215 (16)	0.0224 (16)	−0.0006 (12)	0.0033 (12)	−0.0073 (13)
O8	0.0237 (16)	0.0220 (16)	0.0209 (16)	−0.0013 (13)	−0.0038 (13)	0.0024 (13)

### Geometric parameters (Å, °)

**Table:**

Er1—O4	2.333 (3)	N3—C10	1.328 (6)
Er1—O7	2.367 (3)	N3—C7	1.341 (5)
Er1—O1	2.385 (3)	N4—C8	1.335 (5)
Er1—O6^i^	2.387 (3)	N4—C9	1.336 (6)
Er1—O8^ii^	2.388 (3)	C1—C2	1.510 (6)
Er1—O2^iii^	2.403 (3)	C2—C3	1.370 (6)
Er1—O5	2.451 (3)	C3—H3	0.9300
Er1—N1	2.611 (4)	C4—C5	1.387 (7)
Er1—N3	2.636 (4)	C4—H4	0.9300
Ag1—N4^iv^	2.299 (4)	C5—H5	0.9300
Ag1—O3^v^	2.312 (3)	C6—C7	1.506 (6)
Ag1—N2	2.368 (4)	C7—C8	1.385 (6)
Ag1—O5^vi^	2.648 (4)	C8—H8	0.9300
O1—C1	1.253 (5)	C9—C10	1.387 (6)
O2—C1	1.252 (5)	C9—H9	0.9300
O3—C6	1.236 (5)	C10—H10	0.9300
O4—C6	1.265 (5)	C11—O6	1.252 (5)
O5—C11	1.245 (5)	C11—C11^i^	1.537 (8)
O7—C12	1.252 (5)	C12—O8	1.247 (5)
N1—C5	1.332 (6)	C12—C12^ii^	1.549 (8)
N1—C2	1.337 (5)	O1W—H1W	0.86
N2—C3	1.328 (6)	O1W—H2W	0.86
N2—C4	1.332 (6)		
			
O4—Er1—O7	138.16 (10)	C3—N2—C4	115.8 (4)
O4—Er1—O1	84.40 (11)	C3—N2—Ag1	114.5 (3)
O7—Er1—O1	83.98 (10)	C4—N2—Ag1	128.4 (3)
O4—Er1—O6^i^	135.46 (10)	C10—N3—C7	116.3 (4)
O7—Er1—O6^i^	76.17 (10)	C10—N3—Er1	127.9 (3)
O1—Er1—O6^i^	135.36 (10)	C7—N3—Er1	115.7 (3)
O4—Er1—O8^ii^	91.81 (11)	C8—N4—C9	117.0 (4)
O7—Er1—O8^ii^	68.02 (10)	C8—N4—Ag1^ix^	127.4 (3)
O1—Er1—O8^ii^	132.72 (11)	C9—N4—Ag1^ix^	115.6 (3)
O6^i^—Er1—O8^ii^	75.03 (10)	O2—C1—O1	126.4 (4)
O4—Er1—O2^iii^	78.18 (10)	O2—C1—C2	117.4 (4)
O7—Er1—O2^iii^	138.78 (10)	O1—C1—C2	116.2 (4)
O1—Er1—O2^iii^	81.23 (11)	N1—C2—C3	120.9 (4)
O6^i^—Er1—O2^iii^	87.99 (10)	N1—C2—C1	116.7 (4)
O8^ii^—Er1—O2^iii^	143.95 (10)	C3—C2—C1	122.3 (4)
O4—Er1—O5	69.30 (10)	N2—C3—C2	123.2 (4)
O7—Er1—O5	128.53 (10)	N2—C3—H3	118.4
O1—Er1—O5	147.36 (10)	C2—C3—H3	118.4
O6^i^—Er1—O5	66.24 (9)	N2—C4—C5	121.7 (4)
O8^ii^—Er1—O5	69.19 (10)	N2—C4—H4	119.2
O2^iii^—Er1—O5	74.90 (10)	C5—C4—H4	119.2
O4—Er1—N1	139.39 (11)	N1—C5—C4	121.7 (4)
O7—Er1—N1	67.09 (10)	N1—C5—H5	119.1
O1—Er1—N1	64.79 (10)	C4—C5—H5	119.1
O6^i^—Er1—N1	70.67 (10)	O3—C6—O4	126.7 (4)
O8^ii^—Er1—N1	128.36 (10)	O3—C6—C7	117.7 (4)
O2^iii^—Er1—N1	71.78 (10)	O4—C6—C7	115.6 (4)
O5—Er1—N1	125.49 (10)	N3—C7—C8	122.2 (4)
O4—Er1—N3	63.46 (10)	N3—C7—C6	115.2 (4)
O7—Er1—N3	75.12 (11)	C8—C7—C6	122.6 (4)
O1—Er1—N3	65.75 (11)	N4—C8—C7	121.0 (4)
O6^i^—Er1—N3	141.34 (11)	N4—C8—H8	119.5
O8^ii^—Er1—N3	70.51 (11)	C7—C8—H8	119.5
O2^iii^—Er1—N3	130.51 (11)	N4—C9—C10	121.6 (4)
O5—Er1—N3	115.07 (10)	N4—C9—H9	119.2
N1—Er1—N3	119.41 (11)	C10—C9—H9	119.2
N4^iv^—Ag1—O3^v^	121.94 (12)	N3—C10—C9	121.9 (4)
N4^iv^—Ag1—N2	95.91 (13)	N3—C10—H10	119.0
O3^v^—Ag1—N2	106.55 (13)	C9—C10—H10	119.0
C1—O1—Er1	125.8 (3)	O5—C11—O6	127.3 (4)
C1—O2—Er1^vii^	156.2 (3)	O5—C11—C11^i^	116.8 (4)
C6—O3—Ag1^viii^	123.6 (3)	O6—C11—C11^i^	115.9 (4)
C6—O4—Er1	129.8 (3)	O8—C12—O7	127.2 (4)
C11—O5—Er1	119.3 (3)	O8—C12—C12^ii^	116.4 (5)
C12—O7—Er1	118.2 (3)	O7—C12—C12^ii^	116.3 (4)
C5—N1—C2	116.5 (4)	H1W—O1W—H2W	103.0
C5—N1—Er1	128.9 (3)	C11—O6—Er1^i^	121.8 (3)
C2—N1—Er1	114.6 (3)	C12—O8—Er1^ii^	117.8 (3)

Symmetry codes: (i) −*x*+1, −*y*, −*z*; (ii) −*x*+1, −*y*, −*z*+1; (iii) *x*, −*y*+1/2, *z*−1/2; (iv) *x*+1, −*y*+1/2, *z*−1/2; (v) *x*+1, −*y*+1/2, *z*+1/2; (vi) −*x*+1, *y*+1/2, −*z*+1/2; (vii) *x*, −*y*+1/2, *z*+1/2; (viii) *x*−1, −*y*+1/2, *z*−1/2; (ix) *x*−1, −*y*+1/2, *z*+1/2.

### Hydrogen-bond geometry (Å, °)

**Table:**

*D*—H···*A*	*D*—H	H···*A*	*D*···*A*	*D*—H···*A*
O1W—H1W···O1	0.86	2.14	2.971 (6)	162
